# Supplementary material for: Spatial regulation of thermomorphogenesis by HY5 and PIF4 in Arabidopsis
Source: Nat Commun. 2021 Jun 16;12:3656. doi: 10.1038/s41467-021-24018-7 (PMC8209091; doi:10.1038/s41467-021-24018-7)
Supplement: Supplementary file 1 — Supplementary information [file 41467_2021_24018_MOESM1_ESM.pdf]

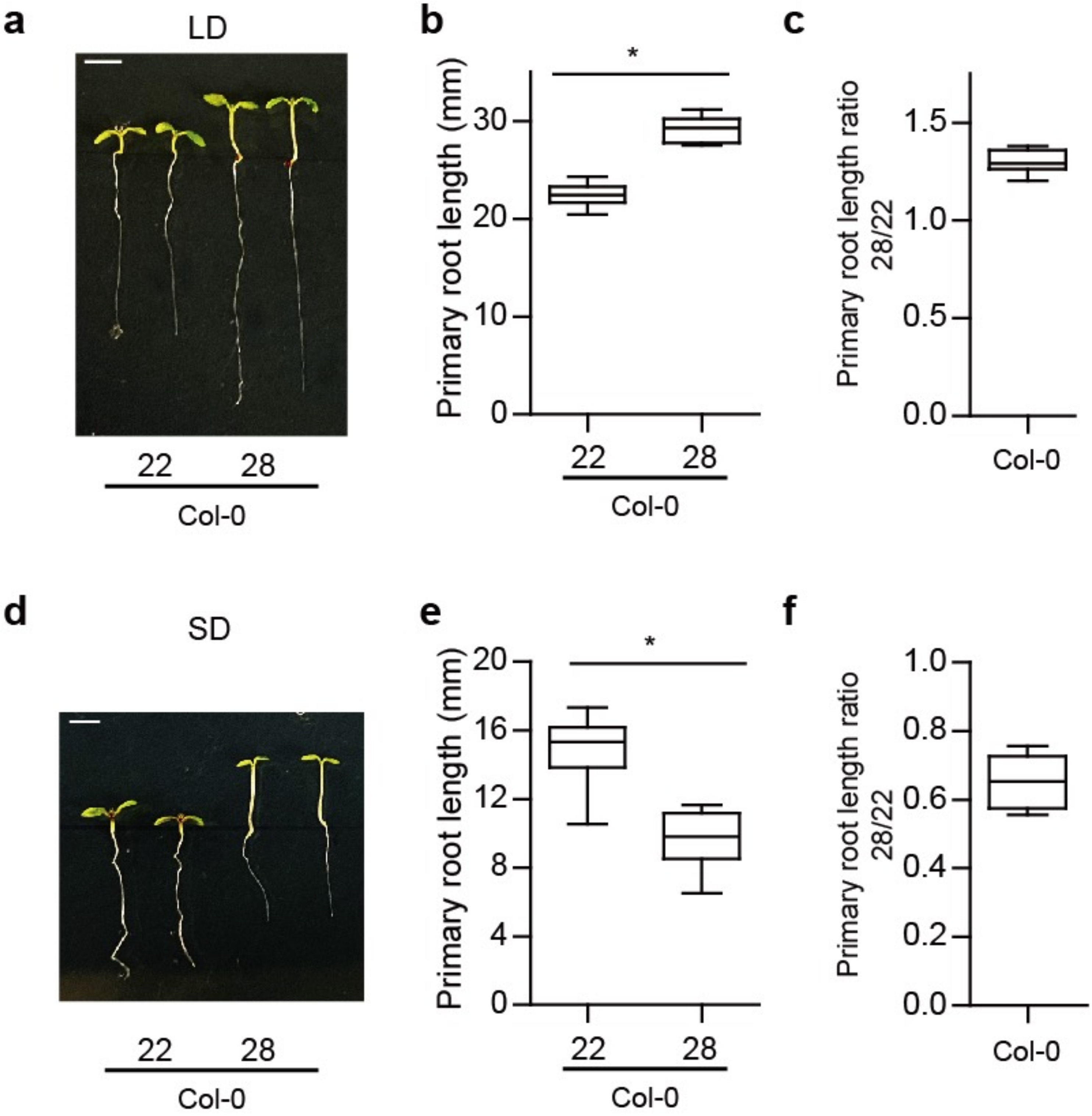

Supplementary Figure 1: Primary root phenotype in long-day (LD) and short-day (SD) at high ambient temperature.

(a, d) Photograph showing seedling phenotypes of Columbia-0 in LD and SD with 22°C and 28°C. Seedlings were grown for two days in continuous white light at 22°C and then transferred to each condition for additional 4 days before being photographed. The scale bar represents 2 mm. (b, c, e, f) Bar graph shows the primary root lengths for seedlings grown under conditions described in (a, d). (c, f) For the length ratio 28/22, hypocotyl length at 28°C is divided by that at 22°C. Asterisks indicate statistical difference using two-sided Student's t-test ($n=10$, $P<0.05$). P values for LD root 22-28: 6.63E-10, SD root 22-28 : 5.76E-6, respectively.

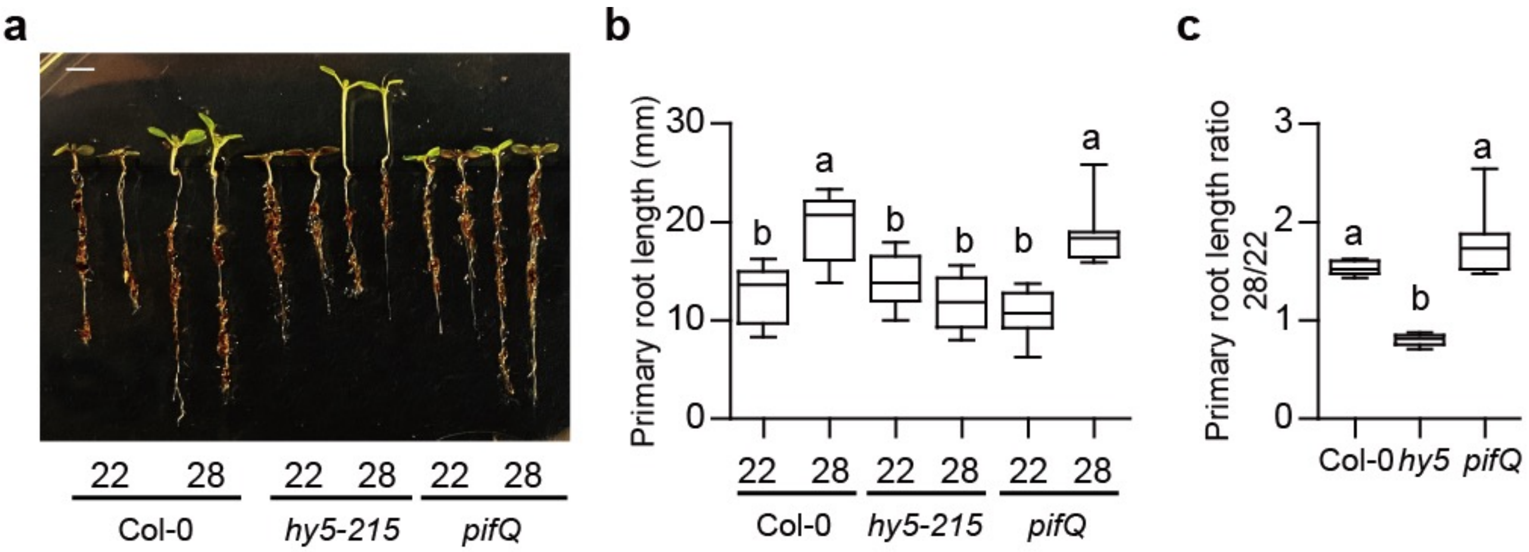

Supplementary Figure 2: Primary root phenotype in the soil under long-day (LD) at high ambient temperature.

(a) Photograph showing seedling phenotypes of Col-0, *hy5-215* and *pifQ* mutants in the soil under LD with 22℃ and 28℃. Seedlings were grown for three days in continuous white light at 22℃ and then transplanted to the soil at 22℃ and 28℃ with cover for one day. Additional four days were treated after removal of the cover before being photographed. The scale bar represents 2 mm. (b) Bar graph shows the primary root lengths for seedlings grown under conditions described in (a). (c) For the length ratio 28/22, hypocotyl length at 28℃ is divided by that at 22℃. Asterisks indicate statistical difference using Student's t-test (n=10, P<0.05).

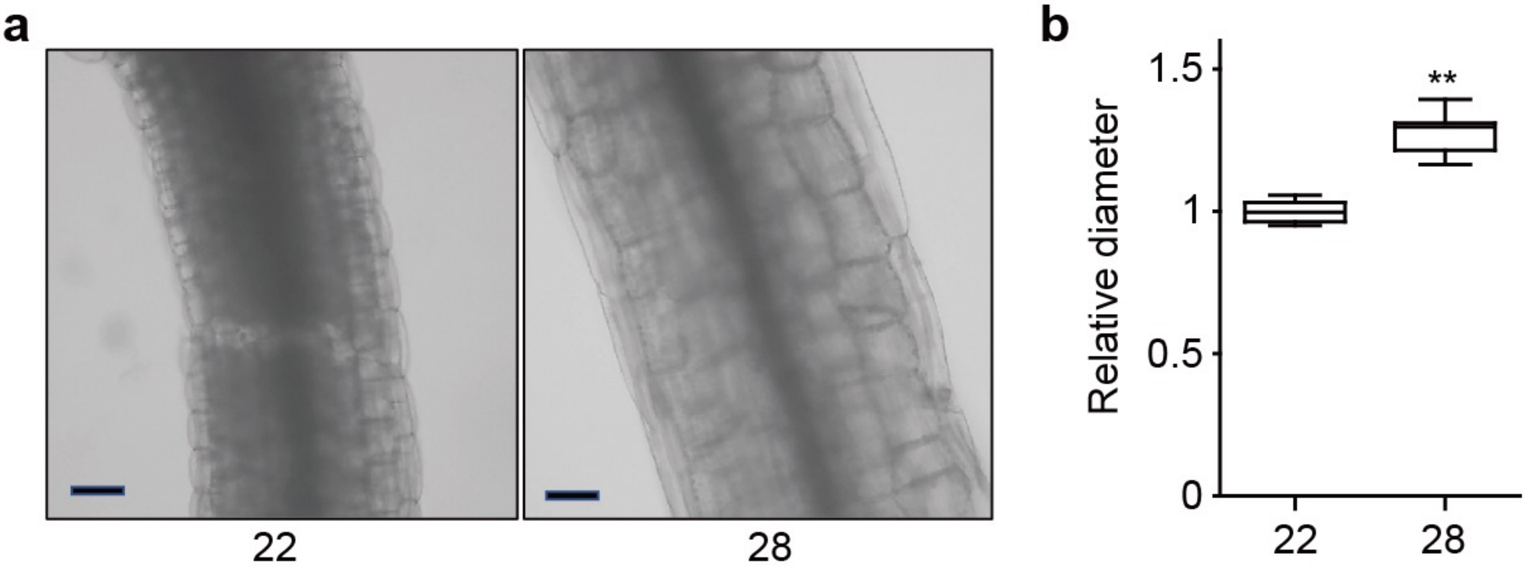

Supplementary Figure 3: Hypocotyl thickness phenotype at high ambient temperature.

(a) Image showing hypocotyl thickness phenotype of Columbia-0 in 22℃ and 28℃. Seedlings were grown for two days in continuous white light at 22℃ and then either kept at 22℃ or transferred to 28℃ for additional 4 days before being imaged. The scale bar represents 200 ㎛. (b) Bar graph shows the relative diameter, dividing hypocotyl diameter in 28℃ to 22℃ described in (a). Asterisks indicate statistical difference using two-sided Student's t-test (n=10, P<0.01). P value: 3.65E-8.

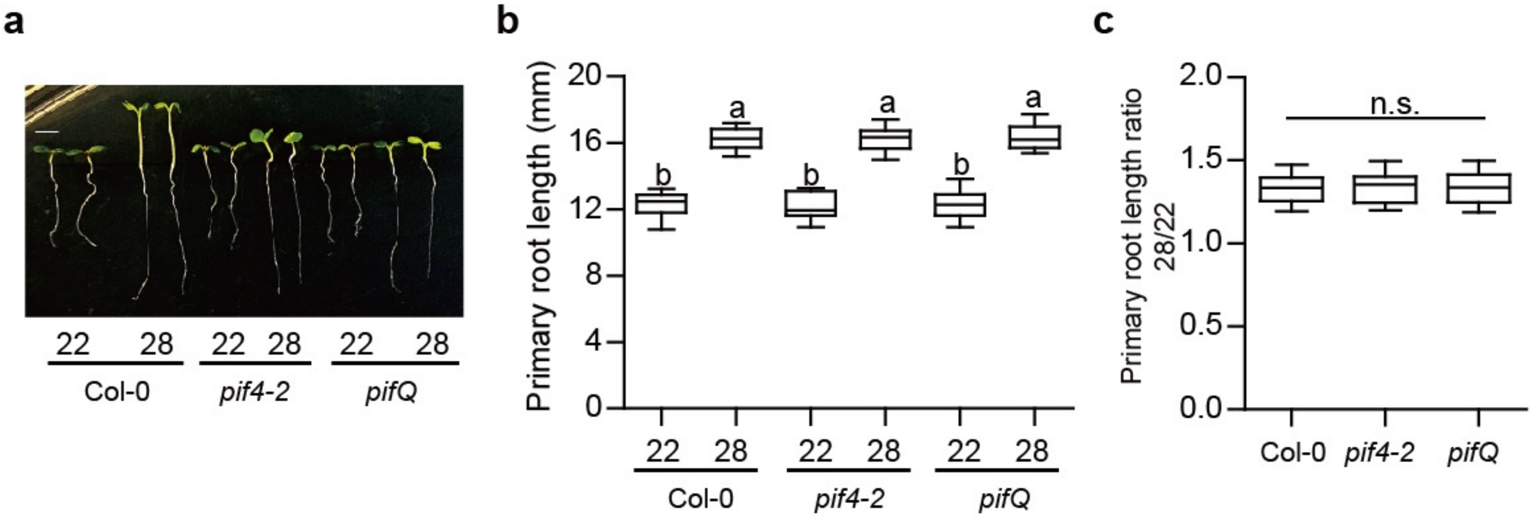

Supplementary Figure 4: PIFs are not involved in thermo-induced primary root elongation.

(a) Photograph showing seedling phenotypes of Columbia-0, *pif4-2* and *pifQ* in 22°C and 28°C. Seedlings were grown for two days in continuous white light at 22°C and then either kept at 22°C or transferred to 28°C for additional 4 days before being photographed. The scale bar represents 5 mm. (b) Bar graph shows the primary root lengths for seedlings grown under conditions described in (c). The letters a-b indicate statistically significant differences between means of primary root length ($P<0.05$, $n=10$) based on one-way ANOVA analyses with Tukey's HSD test. (c) For the length ratio 28/22, hypocotyl length at 28°C is divided by that at 22°C. n.s. stands for not significant according to two-sided Student's t-test ($P<0.05$).

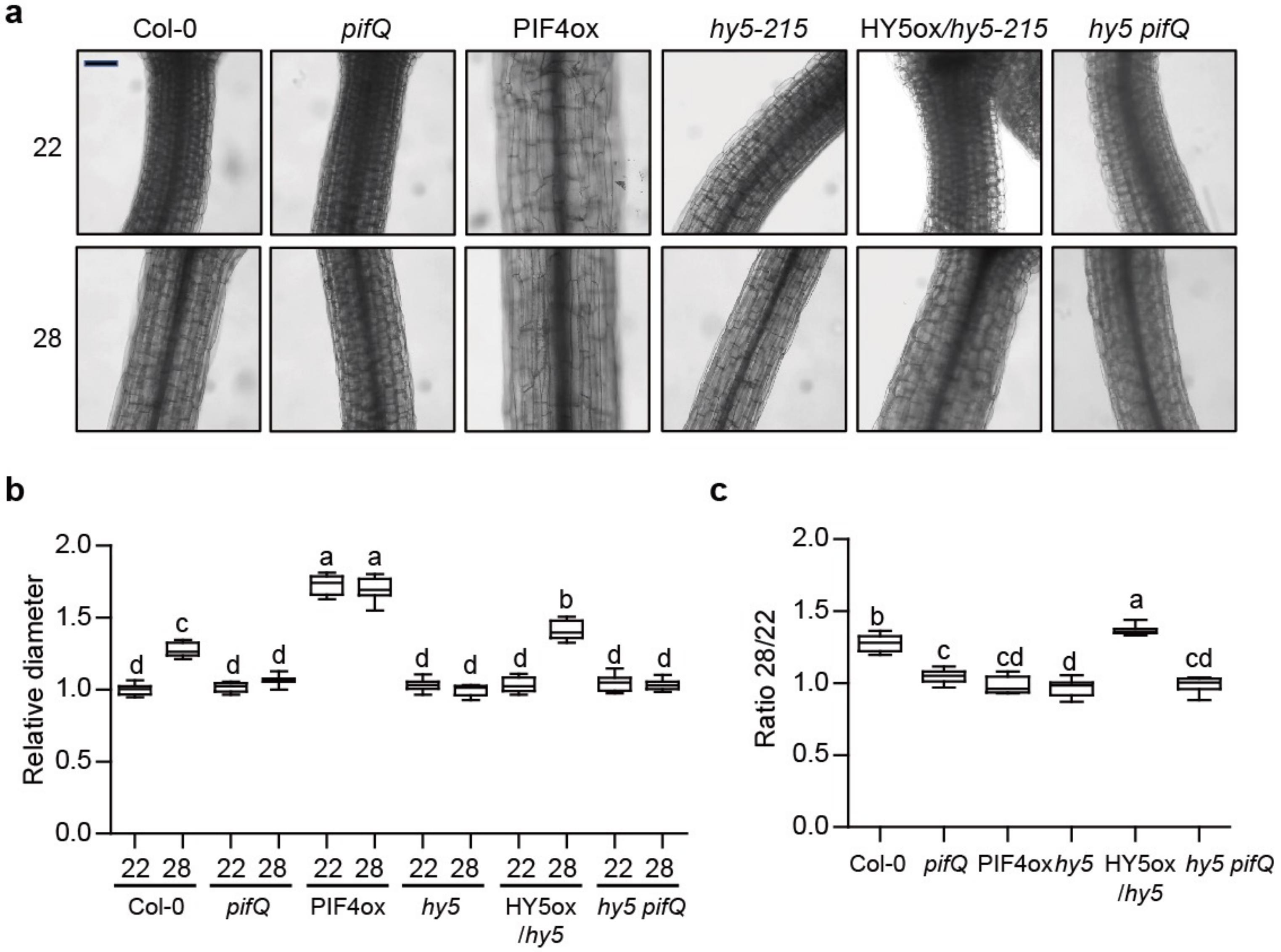

Supplementary Figure 5: Both PIF4 and HY5 are involved in thermo-induced hypocotyl thickening.

(a) Image showing hypocotyl thickness phenotype of Col-0, *pifQ*, 35S:PIF4-myc, *hy5-215*, 35S:HY5-GFP/*hy5-215* and *hy5 pifQ* in 22℃ and 28℃. Seedlings were grown for two days in continuous white light at 22℃ and then either kept at 22℃ or transferred to 28℃ for additional 4 days before being imaged. The scale bar represents 200 ㎛. (b) Bar graph shows the relative diameter, dividing hypocotyl diameter in 28℃ to that of wild-type diameter in 22℃ described in (a). The letters a-d indicate statistically significant differences between means of relative diameter (P<0.05, n=10) based on one-way ANOVA analyses with Tukey's HSD test. (c) For the ratio 28/22, relative diameter at 28℃ is divided by that at 22℃. n.s. stands for not significant according to two-sided Student’s t-test (P<0.05).

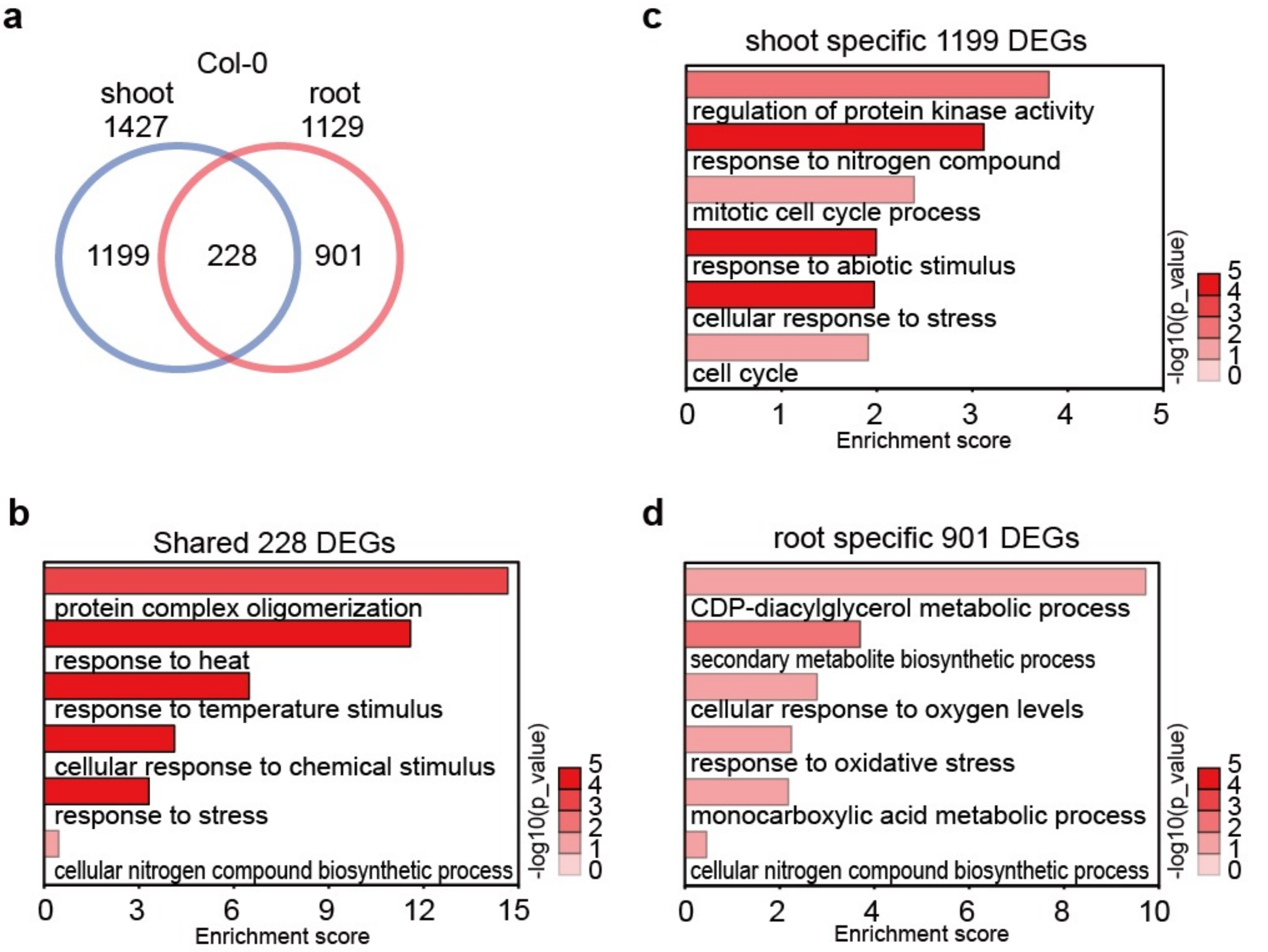

Supplementary Figure 6: Hypocotyl and root show shared but distinct DEGs in thermomorphogenesis.

(a) Venn diagrams show tissue-specific (hypocotyl and root) Differentially Expressed Genes (DEGs) in wild-type (WT) at high ambient temperature response. Six-day-old white light-grown seedlings were transferred to 22℃ or 28℃ for additional 4 hours and total RNA was extracted from three biological replicates for RNA-seq analyses. (b-d) Gene Ontology (GO) analysis of shared 228 DEGs in both tissues (b), hypocotyl specific 1199 DEGs (c), and root specific 901 DEGs (d) at high ambient temperature. Adjusted P-value is from FDR_<0.05 according to the DEG cutoff.

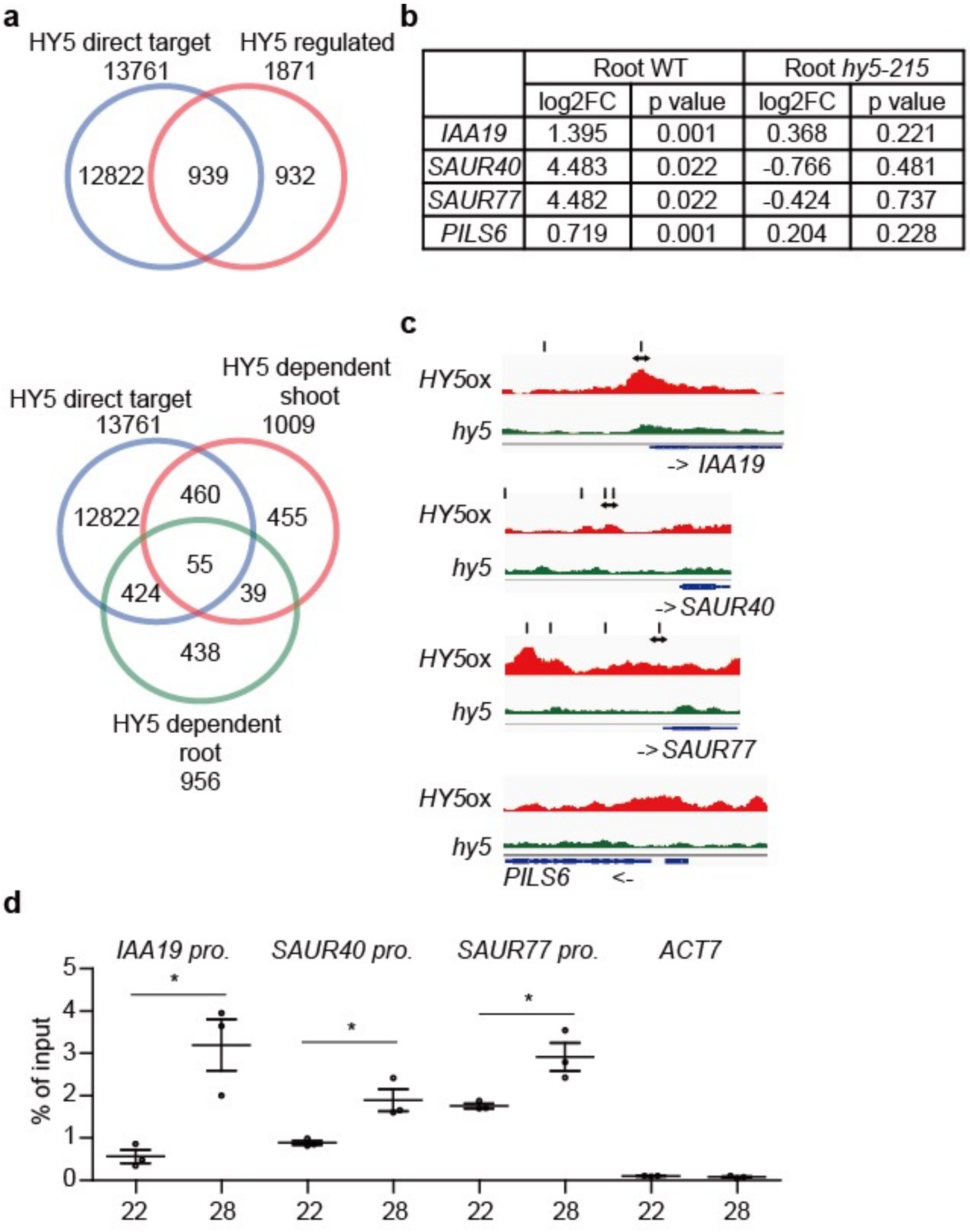

	Root WT		Root *hy5-215*	
	log2FC	p value	log2FC	p value
IAA19	1.395	0.001	0.368	0.221
SAUR40	4.483	0.022	-0.766	0.481
SAUR77	4.482	0.022	-0.424	0.737
PILS6	0.719	0.001	0.204	0.228

Supplementary Figure 7: HY5 direct targets are involved in transcriptional regulation.

(a) Venn diagrams show HY5 direct target and HY5 regulated DEGs (upper panel). Lower panel shows tissue-specific (shoot and root) HY5 regulated DEGs in wild-type (WT) at thermomorphogenesis. (b) RNA-seq data show transcription level of *IAA19*, *SAUR40* and *SAUR77* in WT and *hy5-215* root. Adjusted P-value is from FDR_<0.05 according to the DEG cutoff. (c) Visualization of HY5 binding to the *IAA19*, *SAUR40* and *SAUR77* promoters. Arrowheads indicate the transcription direction of the gene. HY5 direct target and HY5 ChIP-seq data are from Burko et al., 2020. Thick bars indicate the ACGT motif. Arrowlines are the amplified regions from the primers for (d). (d) *IAA19, SAUR40,* and *SAUR77* promoter enrichment of 35S:HY5-GFP/*hy5-215* using Chromatin Immunoprecipitation (ChIP)-qPCR. For ChIP-qPCR, three biological repeats were performed. Error bars indicate SD (n=3). Relative gene expression levels were normalized using input sample. Asterisks indicate statistically significant difference using two-sided Student's t-test; *p<0.05. P values for *IAA19* pro 22-28: 0.013483, *SAUR40* pro 22-28: 0.020326, *SAUR77* pro 22-28: 0.025316, respectively.

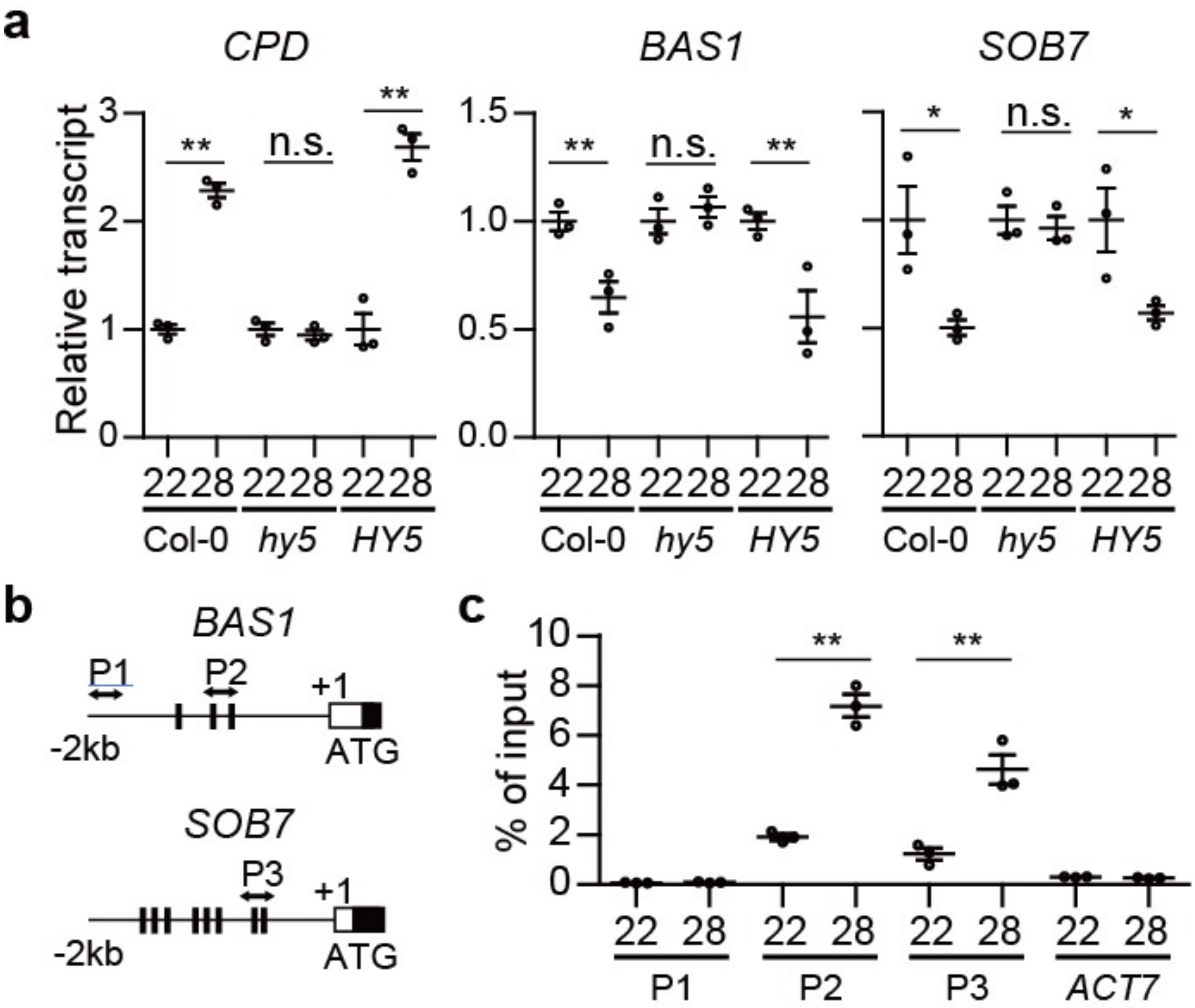

Supplementary Figure 8: HY5 regulates transcriptional level of BR signaling pathway via direct binding to *BAS1* and *SOB7* promoter.

(a) RT-qPCR analysis using *CPD, BAS1, and SOB7*. RT-qPCR samples were from Col-0, *hy5-215,* and 35S:HY5-GFP/*hy5-215* root grown for 5 days at 22℃ and then either kept at 22℃ or transferred to 28℃ for 4 hours. *HY5* stands for 35S:HY5-GFP/*hy5-215*. (b) Simple genomic region scheme of *BAS1* and *SOB7*. Thick bars indicate the ACGT motif. Arrowlines are the amplified regions from the primers. (c) *BAS1* and *SOB7* promoter enrichment of 35S:HY5-GFP/*hy5-215* using Chromatin Immunoprecipitation (ChIP)-qPCR. For RT-qPCR and ChIP-qPCR, three biological repeats were performed. Error bars indicate SD (n=3). Relative gene expression levels were normalized using input sample. Asterisks indicate statistically significant difference using two-sided Student's t-test; *p<0.05 and **p < 0.01. For RT-qPCR, P values for *CPD* in Col-0 22-28: 9.07E-5, in *HY5* 22-28: 0.000884, *BAS1* in Col-0 22-28: 0.013816, in *HY5* 22-28: 0.024882, *SOB7* in Col-0 22-28: 0.034931, in *HY5* 22-28: 0.046244, respectively. For ChIP-qPCR, P values for P2 22-28: 0.00504, P3 22-28: 0.01827, respectively.

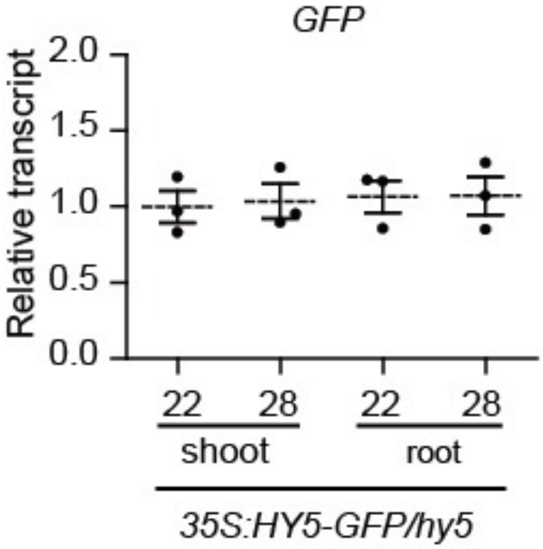

Supplementary Figure 9: Relative transcript level of *HY5-GFP* in shoot and root.

qPCR was performed to detect *HY5-GFP* transcriptional level. Shoot and root samples were from *35S:HY5-GFP/hy5-215* whole seedling grown for 5 days in 22℃ and transferred to 22℃ or 28℃ for 4 hours. Three biological replicates were used in this study. Error bars indicate SD (n=3). Relative gene expression levels were normalized using expression levels of *ACT7.* n.s. stands for not significant according to Student's t-test ($P<0.05$).

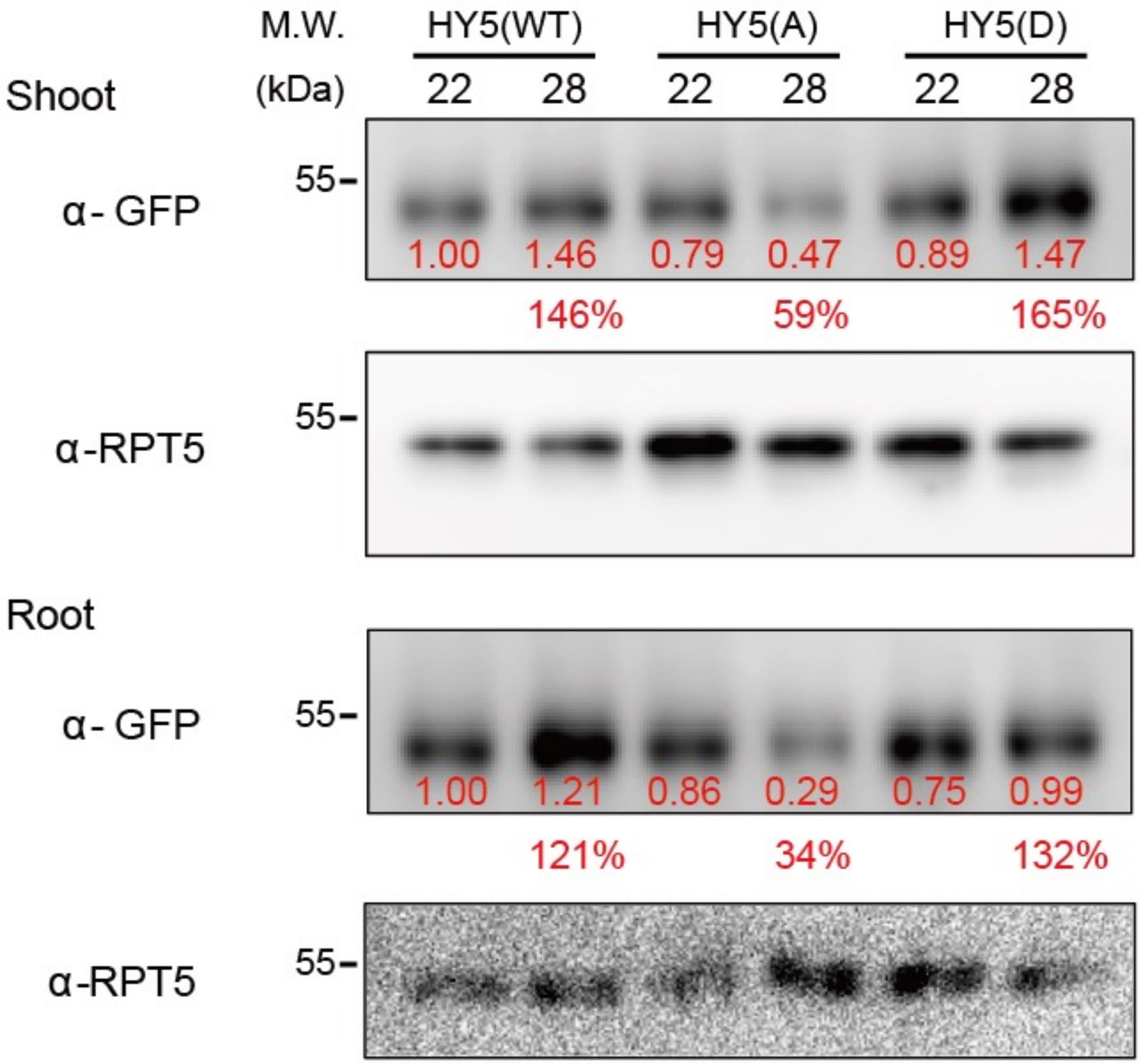

Supplementary Figure 10: HY5-GFP level of 3 different types of HY5 overexpression line.

Western blot shows the level of HY5-GFP from 35S:HY5-GFP/*hy5-215* shoot and root grown for 5 days in 22℃ and either kept at 22℃ or transferred to 28℃ for 4 hours. HY5-GFP was detected using GFP antibody. HY5(WT); 35S:HY5-GFP/*hy5-215*, HY5(A); 35S:HY5 S36A -GFP/*hy5-215,* HY5(D); 35S:HY5 S36D -GFP/*hy5-215.* Red number indicates the quantitation value from anti-GFP band divided by the anti-RPT5 band intensity relative to 22 ℃ of HY5 (WT). Red number in percentage indicates the ratio between 28/22 from the quantitated value from anti-GFP/anti-RPT5 relative to 22 ℃ condition of each line. Two independent experiments were repeated with similar results.

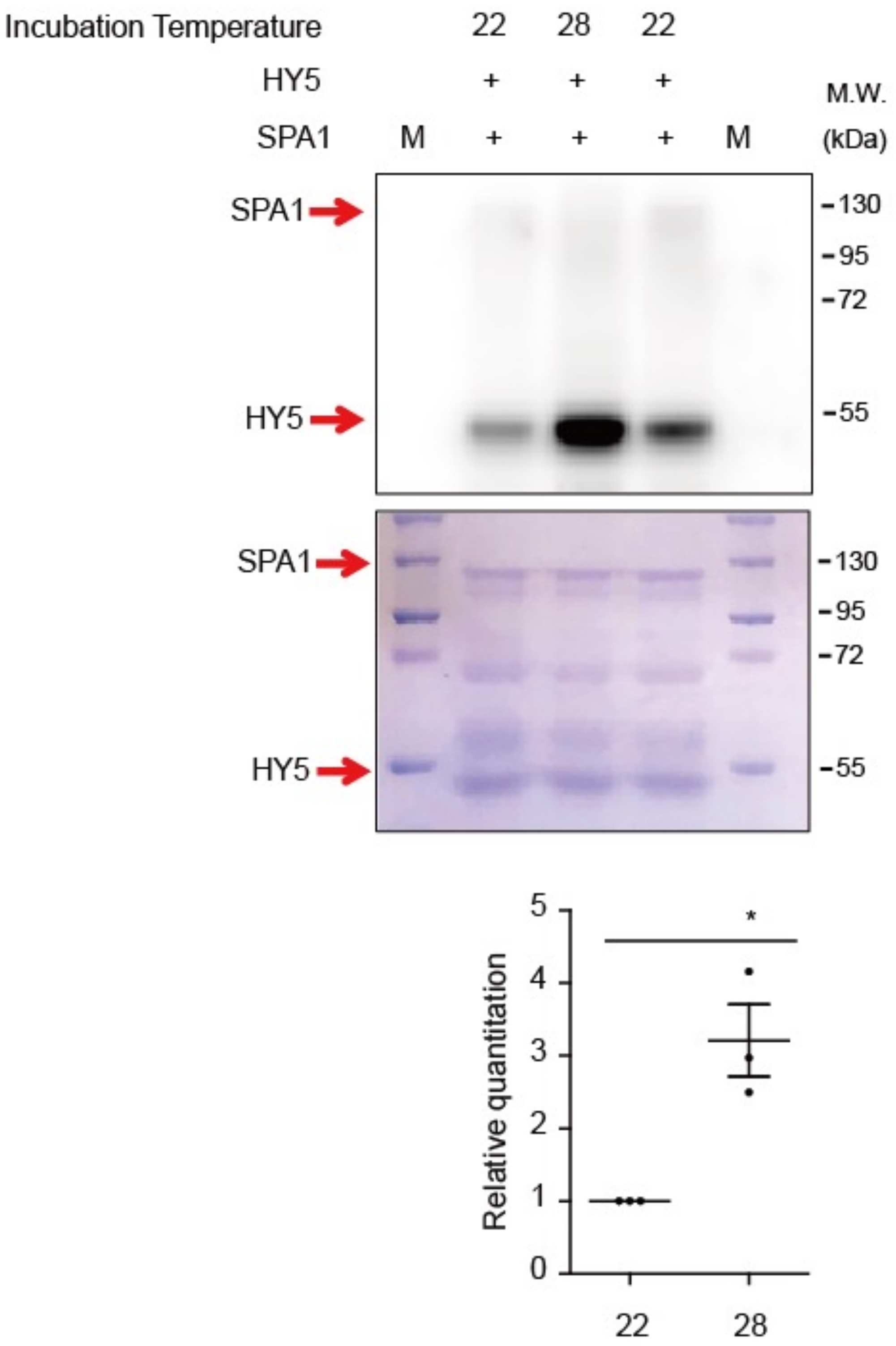

Supplementary Figure 11: SPA1 kinase activity is regulated by high ambient temperature *in vitro*. (Top) Autoradiogram shows SPA1 kinase activity on HY5 at 22℃ and 28℃, respectively. *In vitro* kinase assay was performed using purified SPA1-GFP protein purified from *Sacharomyces cerevisiae* and GST-HY5 protein purified from *E. coli*. (Middle) Coomassie stained gel shows the amount of SPA1 and HY5 used for the assay. (Bottom) Line graph indicates the quantitation value from the autoradiograms divided by the Coomassie staining SPA1 band intensity based on three replicates. The ratio of the autoradiograms at 22℃ was set to 1. Error bars indicate SD (n= 3). The median value was used for the measurement of center. The asterisk indicates a significant difference between 22℃ and 28℃ based on two-sided Student's *t*-test ($P < 0.05$). P value : 0.0111.

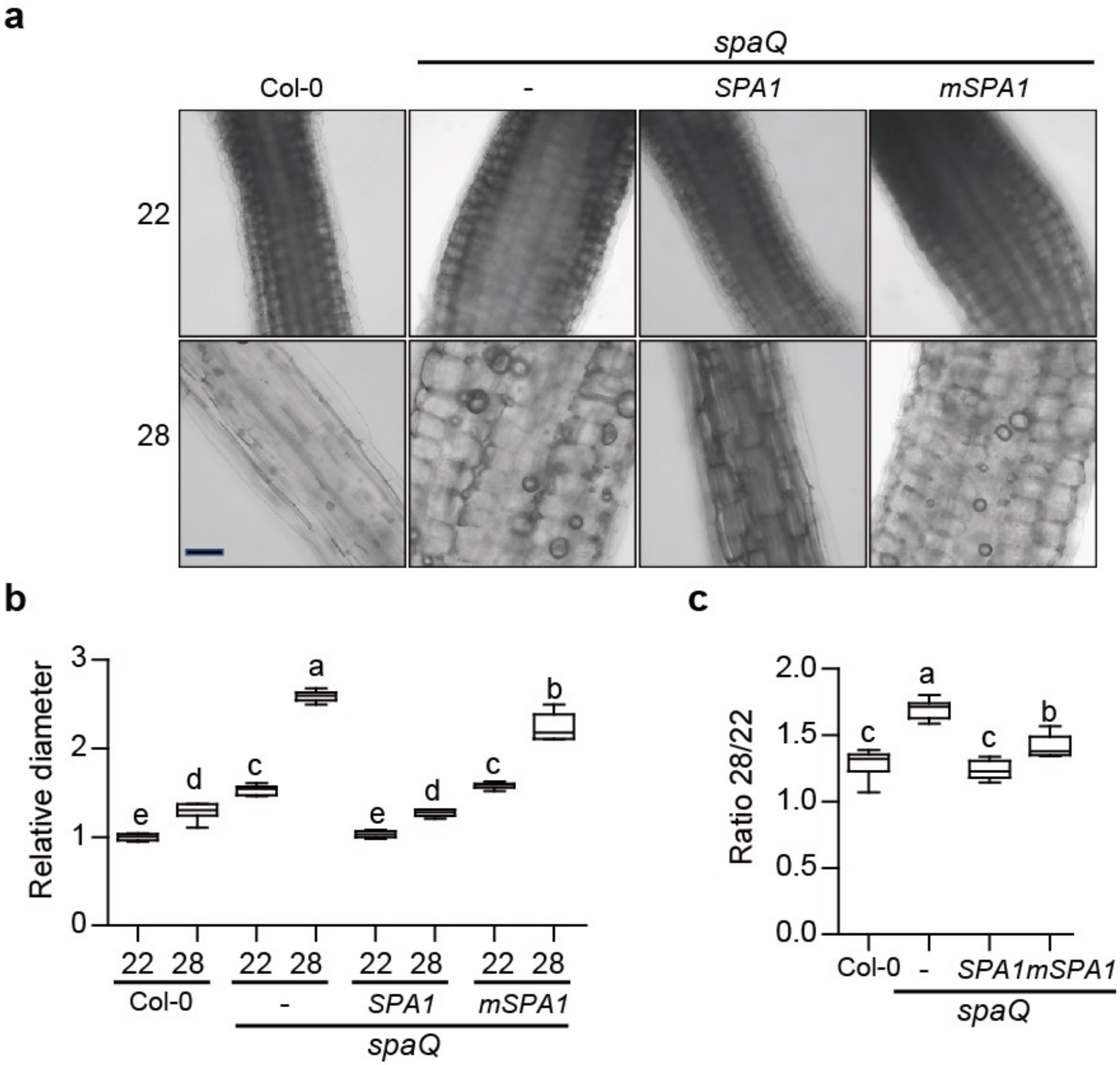

Supplementary Figure 12: SPA is involved in thermo-induced hypocotyl thickening.

(a) Image showing hypocotyl thickness phenotype of Col-0, *spaQ, 35S:SPA1-LUC/spaQ and 35S:mSPA1/spaQ* in 22℃ and 28℃. Seedlings were grown for two days in continuous white light at 22℃ and then either kept at 22℃ or transferred to 28℃ for additional 4 days before being imaged. The scale bar represents 200 ㎛. (b) Bar graph shows the relative diameter, dividing hypocotyl diameter in 28℃ to that of wild-type diameter in 22℃ described in (a). (c) For the ratio 28/22, relative diameter at 28℃ is divided by that at 22℃. The letters a-e indicated in B and C show statistically significant differences between means of relative diameter ($P<0.05$, $n=10$) based on one-way ANOVA analyses with Tukey's HSD test.

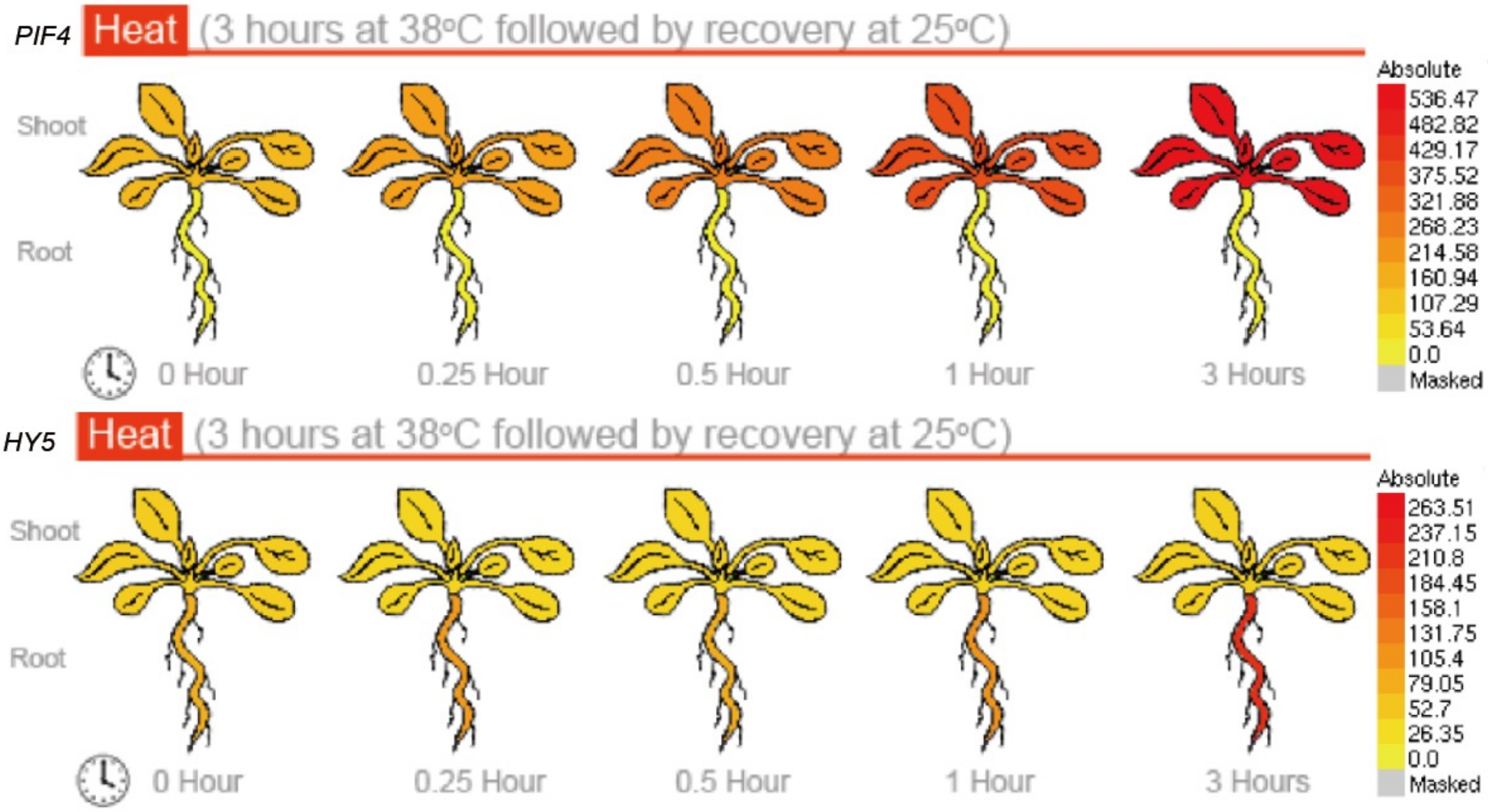

Supplementary Figure 13: *PIF4* and *HY5* show tissue-specific expression under heat stress.

eFP browser data (http://www.bar.utoronto.ca/efp/cgi-bin/efpWeb.cgi) show that *PIF4* and *HY5* display tissue-specific expression in response to heat. (kilian et al., 2007; **https://doi.org/10.1111/j.1365-313X.2007.03052.x**)

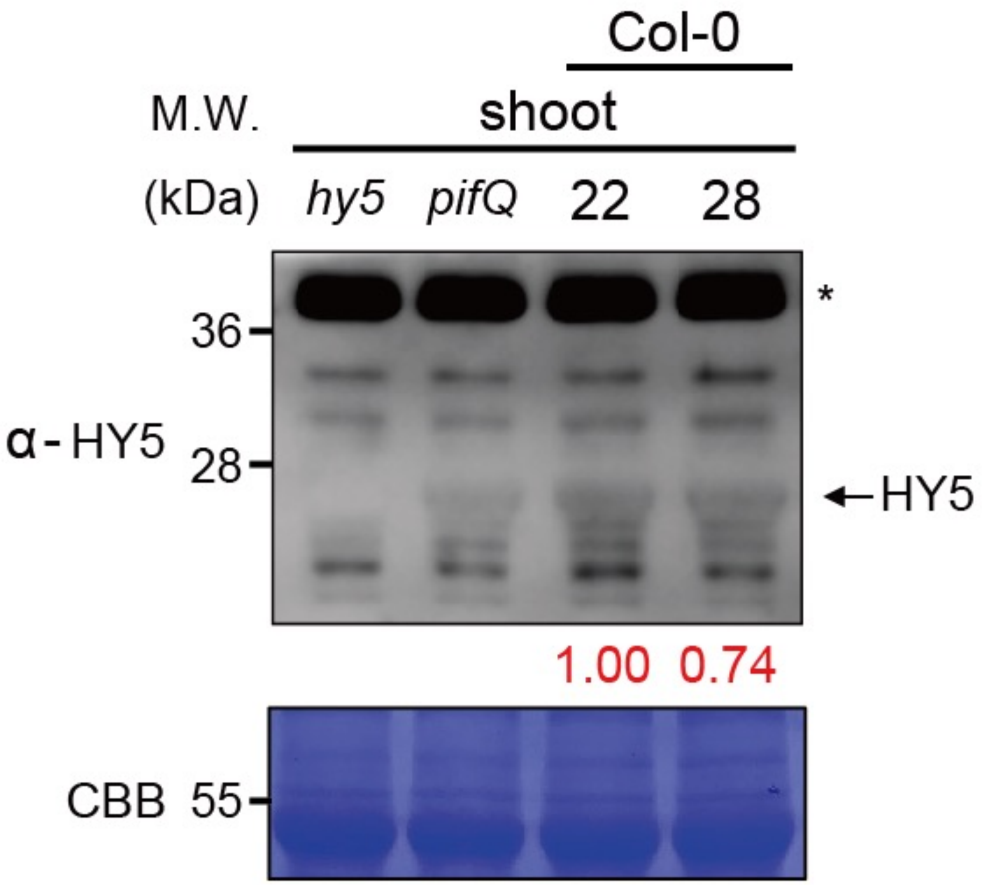

Supplementary Figure 14: The expression of HY5 in the shoot.

Western blot shows the level of native HY5 in the shoot samples separated from whole seedlings grown for 5 days at 22℃ and then either kept at 22℃ or transferred to 28℃ for 4 hours. . *hy5-215* and *pifQ* were used as negative controls. * indicates non-specific cross-reacting bands. Red numbers indicate the relative values for HY5 band divided by the Coomassie staining (CBB) as a loading control. Western blot of HY5 is higher intensity blot of Figure 6C. Two independent experiments were repeated with similar results.

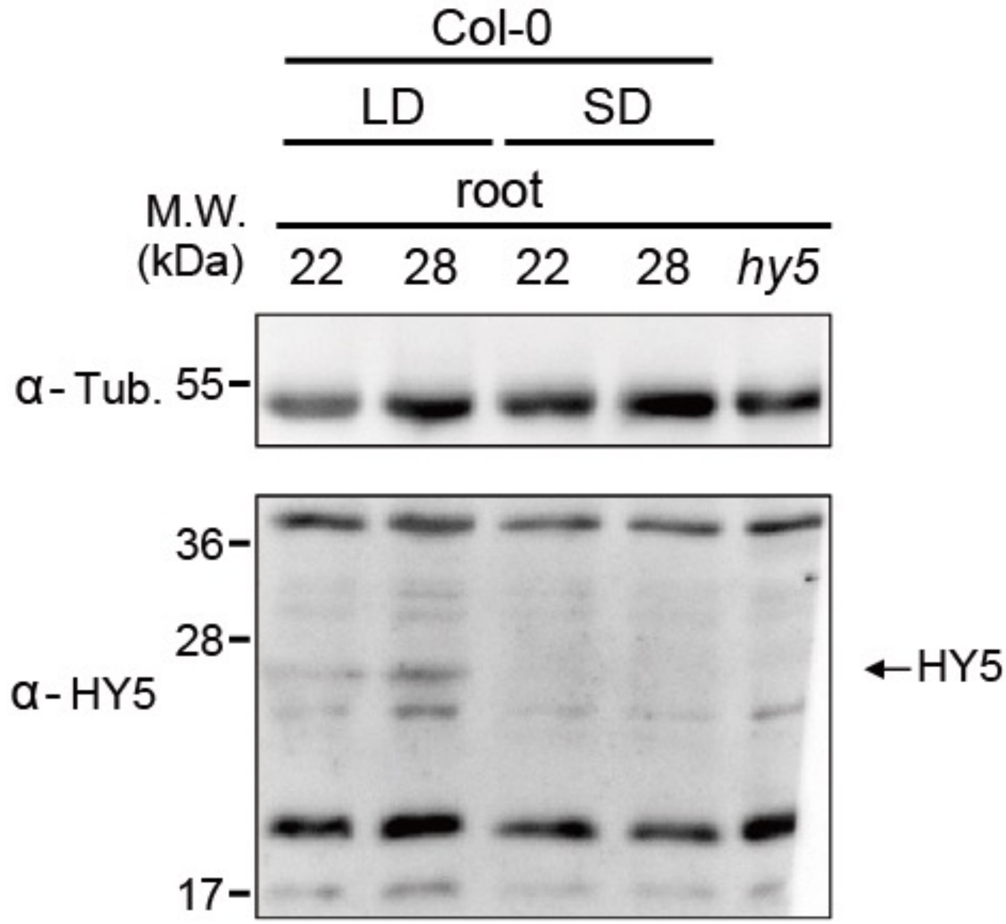

Supplementary Figure 15: The expression of HY5 in the root under LD and SD conditions. Western blot shows the level of native HY5 in the root samples separated from whole seedlings grown for 5 days at 22°C and then either kept at 22°C or transferred to 28°C for 4 hours. Samples were grown under LD or SD conditions. . *hy5-215* was used as a negative control. Alpha tubulin was used as a loading control. Two independent experiments were repeated with similar results.

Supplementary Table 1: Primers used in this study.

Oligonucleotides	
PIF4-F ATGATGTCAATGGGATGTGGAATGAT	This study
PIF4-R GTGGTCCAAACGAGAACCGTCGGTGGTC	This study
HY5-F GCTGCAAGCTCTTTACCATC	This study
HY5-R TCCGACAGCTTCTCCTCCAAACTC	This study
GFP-F GTGAAGGTGATGCAACATACGG	Han et al., 2011
GFP-R AAGTCGTGCCGCTTCATATGA	https://doi.org/10.1073/pnas.1318532111
CPD-F TTGCTCAACTCAAGGAAGAG	Martins et al., 2017
CPD-R TGATGTTAGCCACTCGTAGC	Martins et al., 2017
BAS1-F CCAAGGACCATGTCGTTAAGC	Martins et al., 2017
BAS1-R CCTGAAGTATAGCAAGATTCTGACC	Martins et al., 2017
SOB7-F GTCAGCAAAGAACTAAAGAATCC	Martins et al., 2017
SOB7-R GCTCGAATAGCAAGGAGACC	Martins et al., 2017
SOB7-ChIP-F ATTATTATATCATATATCACATGCG	This study
SOB7-ChIP-R CATGCATGTGGCTTTATTGCATGAG	This study
BAS1-ChIP1-F CTACTACCTCAAGTGTGGAATATG	This study
BAS1-ChIP1-R ATTATACGAGATTAAATGACGGTT	This study
BAS1-ChIP2-F CCATATACGTTACATTGTGAAAAT	This study
BAS1-ChIP2-R TTTCATATGATTGTGATACAAGTC	This study
ACT7-F TCCATGAAACAACTTACAACTCCATCA	{Sun, 2013}
ACT7-R CATCGTACTCACTCTTTGAAATCCACA	{Sun, 2013}
IAA19-F GAACATTTTTCCTATTCATGTTATAT	{Sun, 2013}
IAA19-R ACATATGTTTCTACACACATATATA	{Sun, 2013}
SAUR40-F TCTTCGCCTACGTTGACGGGAACGT	This study
SAUR40-R CTCTAGCATTTTTGGCTGTCTAGGA	This study
SAUR77-F GTTGTCCTACTTGCCTTTCTAGTGG	This study
SAUR77-R TATTTTATTATTTTATTAAAAACCA	This study
IAA19-ChIP-F ATTATTATATCATATATCACATGCG	This study
IAA19-ChIP-R TTCTTGAACTTCTTTTTTTCCTCTC	This study
SAUR40-ChIP-F ACTATTCCAAAAATCTCGACATCTC	This study
SAUR40-ChIP-R GAACACAAAATTAATGTTCATCTTT	This study
SAUR77-ChIP-F ACAAGAGTAGATTGTCCAGCCCACA	This study
SAUR77-ChIP-R AGTATTGGGTTCTTTGTATTTATAT	This study
