# Supplementary material for: Spatial regulation of thermomorphogenesis by HY5 and PIF4 in Arabidopsis
Source: Nat Commun. 2021 Jun 16;12:3656. doi: 10.1038/s41467-021-24018-7 (PMC8209091; doi:10.1038/s41467-021-24018-7)
Supplement: Supplementary file 2 — Descriptions of Additional Supplementary Files [file 41467_2021_24018_MOESM2_ESM.pdf]

Descriptions of Additional Supplementary Files

Supplementary Data 1
Description: List of differentially expressed genes.
